# Condensed Report of the Address of Prof. Agassiz at the Inauguration of the State Geological Hall

**Published:** 1856-09

**Authors:** 


### *Condensed Report of the Address of Prof. Agassiz at the Inauguration of the State Geological Hall.*

### NATURE AN INTELLIGENT WHOLE.

We are assembled to inaugurate the Geological Hall of the State of New York, an institution which has grown out of the State Geological Survey. It has been announcod that Gov. Seward and Hon. Francis E. Gray, would address you upon this occasion. Public duties, however, have detained the one at Washington, and severe sickness prevents the attendance of the other.

I appear here without preparation, and upon an occasion, to me, unusual. But I will endeavor to set before you some of the inducements which exist for communities to patronize and encourage such institutions as this whose opening we meet to celebrate. The occasion is one of high interest. It will hereafter be a marked event in history. The results attained by it are such as have not only called forth our commendation, but have earned the admiration of the whole scientific world.

The Geological Survey of New York has given a new nomenclature to science. Hereafter no geologist can venture to bring his theories before the world, unless he has first consulted the beautiful volumes which embrace its investigations and their results. Already has its fame spread over Europe. When European men of science come to this country, their first question is, "Which is the way to Albany?" "We want to see that State collection of which we have so often read."

What then are the benefits arising from this survey, which elicit this deep interest? The geologists have not found coal. What have they done? They have done more than that. They have gone deeper, and brought out things more valuable. They have taught the wcrld new lessons of admiration and gratitude to its Creator.

Ancient philosophers studied only morals. Then they took upon speculations of astronomy and of physics. Only recently has philosophy turned its attention to the study of plants, of animals and of the crust of the earth. These studies led them irresistibly to the conclusion that Nature can only be the work of an intellectual being,—of Mind,—of an Individual God.

Everywhere there is diversity among organized beings. Everywhere we find types among them that are identical. The two facts, taken together, show that all organized beings have been ordered according to a plan. Thought is visible everywhere: in geological distributions, in organic structure and gradation. Everywhere there is an intellectual connection running through the whole.

Were we not intellectual beings, allied by the nature of our intellect to the Maker of these works, we could not read them. That we can trace the plan, is proof of our mental affinity to the Being that planned them.

For an illustration of this universally appearing plan, take the human arm. It has an upper socket, next a large single bone, next two smaller bones, next the smaller bones of the wrist, next the diverging bones and joints of the hand and fingers. Now take any animal that walks, or creeps, or runs, that has limbs, and you will find the same bones in the same consecutive arrangement. Even the fish, unlike as it appears to a human being, has in its fins what might be a copy of the bones of the human arm. This chain of resemblances shows that one intellect controlled the whole, and ordered them alike. Why should they all be constructed—how could they all be constructed on the same plan, unless they were constructed by the same hand?

The same resembling adaptation of means to ends we find throughout all created animals and plants. Their diversity is in special expressions, their unity in general design.

A fish and a bird, unlike as they look, have the same general anatomical structure. There is the vertebral column, there are the bones diverging from it, there are the cavities above and below, in each. Nay, more. Thousands of fish and birds, thousands of snakes, turtles, of quadrupeds, and so on up to man himself, all are alike in these particulars.

Look at the lizard. There are a vast number of lizards distributed over the globe, differing from each other mainly in the number of their legs. One kind has none. Another has hind legs only. Another fore legs only. Another both. One has a single toe, another two, another three, another four, another five. When brought together in a museum, it is evident that they are variations of the same great family. But to find them you must go all over the world. For one kind you must go to Bengal, for another to Australia, for a third to the Phillipine Islands, for a fourth to South Africa, for a fifth to the Cape of Good Hope, for a sixth to South America, for a seventh to Europe, for an eighth to the United States. They are scattered about the earth, wide as the poles apart, and yet they form, when brought together, a system that we read at a glance! How else could they have been thus formed, unless by an Omniscient, Omnipotent, Provident Creator?

The development of animal life from infancy to maturity, shows the same working of a single intellect. This development during the lifetime of one individual corresponds closely to the gradations from lowest to highest, of the whole series to which the individual belongs. Thus in one series of animals, we have lowest the worm, next above it the crustacea, such as crabs and lobsters, with partially developed legs and head, and next above that insects with perfect head and six legs fully formed. Now, how does the insect develop? Why, in its first stage, *it* is a worm or caterpillar. In the next it is a chrysalis closely resembling the crustacea. In the third it is a perfect insect. It goes through just as many gradations in its lifetime, as there are gradations below it in existence. Here, then, is thought, but thought reaching the same result, through two different processes, in two different series.

Just so the animals of former ages were different from those of the present one, and the whole series has been gradually developed on similar principles. Just so the crustacea now existing, exactly resemble, in their different stages of growth the different and successive kinds of fossil crustacea found in geologic beds. The crab when but a germ, is like a trilobite, the oldest fossil found. As it goes on to maturity it passes through stages, each of which resembles another and another fossil, found in succession, each more complex than the one preceding.

In the vegetable kingdom, the same principle holds. Leaves form regular series. They are arranged according to a regular succession of numbers or fractions. Consider a blade of grass. Its leaves spring alternately on either side. Commencing at the bottom of the stalk and going up spirally, you find the second leaf on the opposite side from the first, the third on the same side as the first, and exactly over it; the fourth over the second, and so on. You go spirally half way round from one to the other.

Now take marsh grass. Its blades are arranged around the stalk in the same way, but the distances are different. The second blade is one-third of the way around the stalk from the first. The next is two-thirds of the way around, and so on.

Take now a rosebush stem. The second leaf is distant from the first, two-fifths of the way around the stalk. The others follow each two-fifths farther around, until finally the sixth is just over the first.

Take again a pine tree twig. The second blade is distant from the first three-eighths of the way around the stalk. The others follow, each three-eighths farther around, until finally the ninth blade is exactly over the first.

Other plants have their leaves arranged each distant the other five-thirteenths of the way around the stalk.

So that we have a series of fractional distances thus:

$\frac{1}{2}$ $\frac{1}{3}$ $\frac{2}{5}$ $\frac{3}{8}$ $\frac{5}{13}$

These fractions, it will be seen, do not differ much from each other. They are none of them less than $\frac{1}{3}$, and none of them more than $\frac{1}{2}$. They form a regularly ascending series, in which any two added together will make the third. Such is the uniform and careful arrangement of the countless leaves of the elms above our heads, and of the pine forests of yonder plains!

Turn now from plants to planets. Measure the time in which each of them circles the sun. It is here:

| | |
|---|---|
| Neptune, - - - - - - | 62,000 days. |
| Uranus, - - - - - - - | 31,000 " |
| Saturn, - - - - - - | 10,000 " |
| Jupiter, - - - - - - - | 4,500 " |
| Asteroids, - - - - - - | 1,600 " |
| Mars, - - - - - - - | 680 " |
| Earth, - - - - - - | 365 " |

Now examine these sums. The second is half the first; the third is one-third of the second; the fourth is two-fifths of the third; the fifth is three-eighths of the fourth; the sixth is five-thirteenths of the fifth. So that we again [illegible] precisely the same fractions in the same order:

$\frac{1}{2}$ $\frac{1}{3}$ $\frac{2}{5}$ $\frac{3}{8}$ $\frac{5}{13}$

Whence this strange similarity? How can it be accounted for except by the fact that the same Hand adjusted the blades of grass which set in motion the orbs of the universe!

